# Effectiveness of orange almond potato cookie vs. orange potato cookie supplementation on nutritional wellbeing of the Indonesian stunted preschool-aged children during COVID-19 pandemic

**DOI:** 10.3389/fnut.2023.1235841

**Published:** 2023-09-25

**Authors:** Fatmah Fatmah, Suyud W. Utomo

**Affiliations:** ^1^Disaster Management Program, School of Environmental Studies at Universitas Indonesia, Jakarta, Indonesia; ^2^School of Environmental Studies at Universitas Indonesia, Jakarta, Indonesia

**Keywords:** preschool-aged children, stunting, COVID-19, Height -for-Age Z-score, cookies

## Abstract

**Background:**

Preschool-aged children who experience stunting due to insufficient consumption of macro- and micronutrients exhibit weakened immune systems, rendering them susceptible to contracting COVID-19 during the ongoing epidemic. Therefore, it is imperative to implement interventions aimed at enhancing the nutritional status of preschool-aged children by providing them with nutrient-rich food supplements as a preventive measure against illness transmission. The objective of the study was to evaluate the impact of incorporating potato almond orange cookies into the diet on the nutritional status of preschool-aged children who are experiencing stunting.

**Methods:**

A non-randomized pre-post intervention study was done on 42 individuals aged 12–58 months during 4 weeks. The intervention group was provided with almond potato cookies, while the control group was given orange potato cookies. During the study period, educational sessions on balanced nutrition in preschool-aged children with stunting and COVID-19 were provided to the mothers of both groups. The data analysis involved conducting univariate and bivariate analyses, namely utilizing the independent *t*-test.

**Results:**

The intervention group exhibited the most significant enhancements in Height-for-Age Z-score (HAZ). The mean Height-for-Age Z-score of the intervention group increased by 0.51 (from −3.15 to −2.64), whereas the control group saw a smaller gain of 0.25 (from −2.69 to −2.44). This increase was influenced by the mother’s age; mother’s education; father’s occupation; family size; good sanitation facilities; healthy home environment; and fat, calcium, and zinc intake from the cookies (*p* < 0.05). From the perspective of knowledge about balanced nutrition and COVID-19, there was no significant difference in the Height-for-Age Z-score in the intervention group.

**Conclusion:**

The ingestion of orange almond potato cookies has the potential to enhance the nutritional wellbeing of children in the preschool age group who are experiencing stunted growth.

## Introduction

The impact of the COVID-19 pandemic on preschool-aged children who are stunted is more pronounced, evidenced by an increased susceptibility to mortality. This vulnerability can be attributed to compromised immunity from inadequate consumption of essential and micronutrients. From 2020 to 2022, there was a notable rise in the prevalence of stunted children globally, attributed to the COVID-19 pandemic, resulting in an estimated increase of 1.6 million cases (1). According to recent data, approximately 22% of children under five exhibited stunted growth in 2020 (2). During the COVID-19 pandemic, a significant number of Indonesian children, namely over 7 million individuals below the age of five, experienced stunted growth (3).

The COVID-19 epidemic has further intensified the issue of food insecurity in Indonesia. Several nations in the Indo-Pacific region, such as Jordan and South Africa, encountered a comparable problem (4, 5). The global occurrence of this condition was anticipated to result in child malnutrition, the interruption of health services, and a decrease in household income, ultimately leading to an increase in child stunting and micronutrient insufficiency. Due to the ongoing pandemic, a significant proportion of households, namely the lower 40%, cannot fulfill the criteria for adequate food consumption as determined by principles of balanced nutrition. The prevalence of undernourishment increased by 8.34%, the consumption of food quality as indicated by the desired dietary pattern increased by 86.3%, and the food insecurity experience scale increased by 5.12% (6).

There exists a synergistic association between stunting and COVID-19 infection. Several factors contribute to stunting, including inadequate maternal nutritional status during pregnancy, insufficient nutrition intake for mothers and children, suboptimal parenting practices, restricted availability of health services, and insufficient access to clean water and nutritious food within the family or community (7). The COVID-19 pandemic has resulted in a drop in food security at the family level, as the purchasing capacity to acquire food has diminished. Consequently, there has been an observed rise in the prevalence of stunting in Indonesia (8).

Addressing stunting has emerged as a prominent agenda in the context of Indonesia. The legal framework for stunting reduction interventions in the Republic of Indonesia has been established by implementing Presidential Decree No. 72 of 2021. This decree categorizes the interventions into two main types: targeted nutritional intervention and sensitive nutritional intervention. The targeted nutritional intervention was designed to enhance both nutrition and health outcomes. The sensitive nutritional intervention encompasses a range of supportive measures aimed at mitigating the occurrence of stunting. These efforts include the provision of clean water and sanitation (9).

Depok City, located in the West Java Province of the Republic of Indonesia, has had an elevated susceptibility to stunting, one of the consequences of the COVID-19 pandemic. Although there has been a decline in the occurrence of stunting among preschool-aged children, with a fall from 6.63% in 2016 to 3.5% in 2021, there was an observed increase in 2020 (12.6%) compared to the prevalence rate in 2019 (4.55%) (10). The rise in stunting among preschool-aged children due to the COVID-19 pandemic poses a challenge to the city’s goal of achieving Zero New Stunting Cases in Depok City (11) and the attainment of Goal No. 2 of the Sustainable Development Goals, which aims to eradicate hunger and malnutrition by 2030 (12). The Depok Government has adopted targeted initiatives to reduce the prevalence of stunting among preschool-aged children during the COVID-19 pandemic. To expedite the attainment of Zero New Stunting Cases in Depok during the COVID-19 pandemic, several innovations were implemented over 3 months to enhance the nutritional wellbeing of preschool-aged children afflicted with stunting (13). The range of sensitive interventions encompasses several strategies aimed at addressing specific needs. These interventions include the implementation of balanced and safe nutritional food diversification programs, initiatives promoting the intake of fish, milk, and eggs, the establishment of sustainable food gardens, mentorship programs in early education institutions, counseling services for prospective brides, and the supply of basic sanitary facilities and clean water (14).

Nevertheless, there persist obstacles in mitigating the prevalence of stunting within this urban area, potentially stemming from inadequate parental practices concerning preschool-aged children, insufficient understanding of nutritionally balanced diets for stunted preschool-aged children, and the disadvantaged socioeconomic circumstances of the parents (15). This situation in Depok warrants nutrition-sensitive interventions in the form of nutritional supplementation for preschool-aged children with stunting. The intervention that involved the provision of orange almond potato cookies as a supplementation in 30 malnourished preschool-aged children in Depok in 2021 resulted in increases in weight (0.4 kg), (1.98 cm), Hb level (0.1 g/dL), and albumin level (0.1 g/dL) (16). Potato starch is selected as the essential ingredient for the cookies for its considerable energy and carbohydrate contents, which will increase (17–19). In addition, almond flour is added due to its vitamin E content, which can increase the leukocyte count. In contrast, with its hesperidin content, marmalade is added for its immunomodulatory, anti-inflammatory, and antioxidant properties (20, 21).

Depok City, located in the West Java Province of the Republic of Indonesia, has had an elevated susceptibility to stunting due to the COVID-19 pandemic. Although there has been a decline in the occurrence of stunting among preschool-aged children, with a fall from 6.63% in 2016 to 3.5% in 2021, there was an observed increase in 2020 (12.6%) compared to the prevalence rate in 2019 (4.55%) (10). The rise in stunting among preschool-aged children due to the COVID-19 pandemic poses a challenge to the city’s goal of achieving Zero New Stunting Cases in Depok City (11) and the attainment of Goal No. 2 of the Sustainable Development Goals, which aims to eliminate hunger and malnutrition by 2030 (12).

The Depok Government has adopted targeted initiatives aimed at reducing the prevalence of stunting among preschool-aged children during the COVID-19 pandemic. In order to expedite the attainment of Zero New Stunting Cases in Depok during the COVID-19 pandemic, a series of innovations were implemented for 3 months to enhance the nutritional wellbeing of preschool-aged children afflicted with stunting (13). The range of sensitive interventions encompasses several strategies to promote wellbeing and address specific needs. These interventions include the implementation of balanced and safe nutritional food diversification programs, initiatives to encourage the intake of fish, milk, and eggs, the establishment of sustainable food gardens, mentorship programs in early education institutions, counseling services for individuals preparing for marriage, and the supply of basic sanitation facilities and clean water (14). Nevertheless, there persist obstacles in mitigating the prevalence of stunting within this urban area, potentially stemming from inadequate parental practices concerning preschool-aged children, insufficient understanding of appropriate nutrition for preschool-aged children afflicted with stunting, and the disadvantaged socioeconomic circumstances of the parents (15).

The present study aimed to evaluate the impact of incorporating orange almond potato cookies into the diets of preschool-aged children with stunting [as indicated by Height -for-Age Z-score (HAZ) in Depok]. The intervention involved providing these cookies to 42 stunted preschool-aged children for 30 days amidst the COVID-19 pandemic. The COVID-19 epidemic that began in 2020 has resulted in a notable absence of literature on nutritional therapy for stunted children in Indonesia. There has been limited scientific research on targeted feeding interventions for malnourished children in the context of the COVID-19 pandemic. A restricted corpus of literature has directed its attention to the significance of these interventions in mitigating the prevalence of malnutrition (22, 23). Therefore, the current study presents a distinct and groundbreaking contribution aimed at improving the nutritional condition of stunted children in Indonesia during the ongoing epidemic.

## Materials and methods

### Study design

This study applied a non-randomized pre-post intervention design (24), focusing on 42 stunted preschool-aged children. The study was conducted in three urban villages, namely Depok, Depok Jaya, and Sawangan, which were selected due to their high prevalence of stunting. These villages are located in Depok City, West Java Province, Indonesia.

### Ethical approval

The Ethics Commission for Health Research and Development (*KEPPK*), affiliated with the Sint Carolus School of Health Sciences, located in Jakarta, conducted the ethical evaluation. The review was completed under the honest clearance number 008/KEPPKSTIKSC/I/2023. In early January 2023, before the commencement of the trial, all mothers of preschool-aged children who met the specified inclusion criteria provided their informed consent by signing the informed consent.

### Population and sample

The study sample consisted of preschool-aged children who had stunted and resided in Depok City throughout the interview. The study sample included preschool-aged children who exhibited stunting and satisfied the specified inclusion criteria. The study included individuals aged 12–59 months, including males and females. The participants resided in the Sawangan Lama, Depok, and Depok Jaya areas of Depok City, three urban villages known for their high prevalence of stunting. The inclusion criteria required individuals to have a Height-for-Age Z-score (HAZ) index below −2 standard deviations and to be free from chronic or infectious disorders. The required sample size was determined using a paired hypothesis test, assuming a 2.0 cm increase in and a standard deviation of 0.1. The present investigation employed a two-tailed significance test with a significance level 0.05. To ensure a confidence interval of 95%, a minimum sample size of 25 was determined (25).

### Sample recruitment

**Figure 1:**
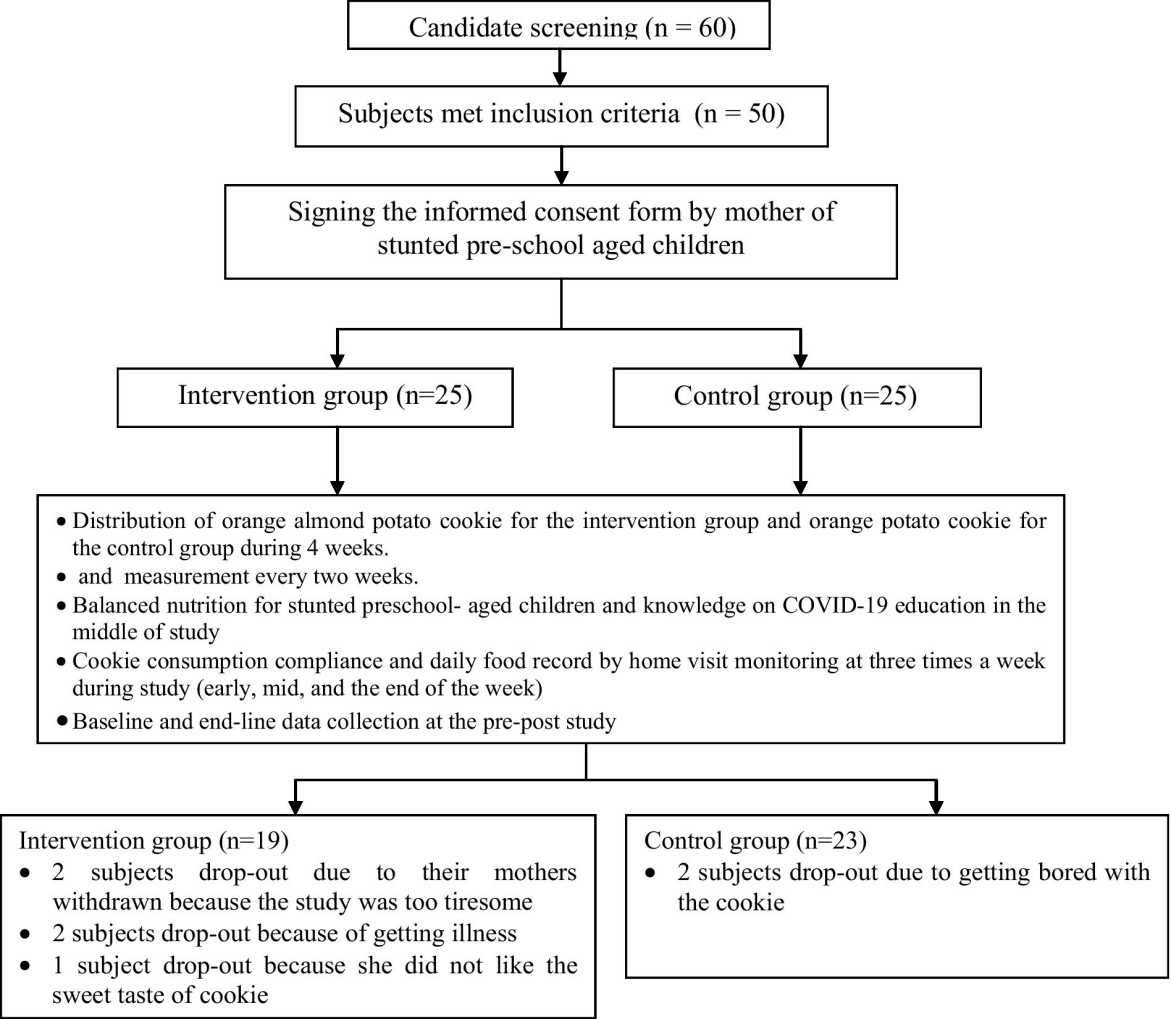
Research scheme.

The study consisted of 42 subjects actively participating in all aspects of the research throughout the 4 weeks. A nutritional status screening was performed on 50 preschool-aged children who fulfilled the specified inclusion criteria. The list of potential subject names was derived from the regular weighing data collected from the integrated health posts (*pos pelayanan terpadu/posyandu*) affiliated with the Pancoran Mas Sub-District Health Center and Sawangan Sub-District Health Center in Depok. The sample consisted of 50 children in the preschool age range assigned to two groups: an intervention group (*n* = 25) that received orange almond potato cookies and a control group (*n* = 25) that received potato cookies without any additional ingredients. During the initial week of the study, six participants from the intervention group discontinued their involvement. The reasons for their withdrawal included two mothers who expressed a desire to withdraw owing to the perceived exertion associated with the study, two subjects who fell unwell, and one participant who disliked the sweet taste of the cookies. Two people from the control group withdrew from the study due to experiencing boredom with the cookie, as depicted in Figure 1.

Various measures were implemented to mitigate potential biases. The study included many methodological strategies to ensure rigor and validity. Firstly, the researchers established clear and defined criteria for the inclusion and exclusion of participants. Secondly, the participants’ cookie consumption was meticulously monitored three times per week utilizing in-home visits conducted by trained enumerators. Lastly, the placebo group was used as a means of comparison for the control group. The output generated by the two groups was subsequently compared.

### Primary data collection

The height was measured using a microtoise and was tested to ensure accuracy. The researchers received a list of preschool-aged children who exhibited stunting from specific sub-district health centers. Additional validation of the nutritional status of preschool-aged children with stunting was conducted at the local *posyandu*. Mothers of preschool-aged children who exhibited stunted growth and fulfilled the specified criteria were sent an invitation to partake in the research dissemination event held at the *posyandu*. In this local community health center, preschool-aged children were enrolled. Simultaneously, the participants were required to sign an informed consent form, which served as the initial page of the questionnaire. The *posyandu* cadres and research team members were present as witnesses during the signing process.

Every group had a daily snack of 50 g cookie supplements for 30 days. It is important to note that this snack was not intended to replace the main meal or rice consumption. Cookies are a popular snack among young individuals because of their crispness and firm consistency. Supplemental feeds are advantageous due to their limited ability to induce prolonged satiety and minimal interference with consuming the primary food supply. Table 1 presents the nutritional composition of orange almond potato cookies, indicating that every 50 g serving of these cookies has 254 calories of energy, 27.7 g of carbohydrates, 3.25 g of protein, 14.4 g of fat, 0.425 mg of zinc, 32.75 mg of calcium, and 2.84 mg of vitamin E.

**Table 1:** Nutrients analysis per 50 g of cookies.

Type of cookies	Energy (kcal)	Carbohydrate (g)	Protein (g)	Fat (g)	Zinc (mg)	Calcium (mg)	Vitamin E (mg)
Orange almond potato	254.0	27.7	3.3	14.4	0.4	32.8	2.8
Orange potato	236.6	33.0	1.4	11.0	0.4	16.4	0.9

The height was assessed bimonthly, and providing educational sessions on nutrition to mothers of preschool-aged children affected by stunting at the same time with the height measurement. The tasks mentioned above were carried out by the three well trained nutrition enumerators utilizing informational materials created by the lead investigator. The total height measurement was obtained thrice throughout the study (at pre, during, and post study). The enumerator collected data on daily food intake through home visits conducted three times each week, specifically at the beginning, middle, and end of each week. A total of 12 days of food recall data were gathered for each child in both groups. The primary objective of conducting the initial and final survey data collection was to acquire a comprehensive understanding of the sociodemographic attributes of the mothers and children involved in the study. Additionally, these data collections aimed to assess the alterations in maternal knowledge on balanced nutrition in preschool-aged children suffering from stunting and changes in maternal knowledge on COVID-19.

The baseline and endline questionnaires included items on sociodemographic characteristics such as maternal age, marital status, and educational background; maternal and paternal occupations; number of people living together in the same household and number of preschool-aged children in the family; age and sex of the children/s; primary caregiver for the preschool-aged children; knowledge of balanced nutrition such as the definition of a healthy food menu; benefits of consuming food as the source for carbohydrates, protein, fat, vitamins, and minerals; familiarity with the term balanced nutrition; knowledge about the need for regular measurement of every month; understanding of the consequences of not measuring the every month; consequences of the lack of gain for 3 consecutive months; impact of not gaining the requisite; knowledge about what to do to increase the of a children; and knowledge of COVID-19, such as familiarity with the term of COVID-19, definition of COVID-19, symptoms of COVID-19, whether a COVID-19 infection can be cured, groups that are vulnerable to COVID-19, mother’s efforts to prevent her children from being infected by COVID-19, and implementation of health protocols for preschool-aged children.

At the onset of the investigation, an evaluation was conducted to analyze the sanitary conditions encompassing the many components of the household, sanitation facilities, and the behavioral patterns exhibited by those residing inside the children’s residential setting. During the baseline questionnaire interview, various aspects of the house were assessed. These included the cleanliness of the ceiling, the condition of the house walls (categorized as permanent, semi-permanent, or not permanent), the type of house floor (soil, boards, or plaster), the presence or absence of bedroom windows, the presence or absence of living room windows, the availability of ventilation, the presence or absence of a kitchen exhaust system, and the quality of lighting (classified as bright, underexposed, or dark). The enumerators, trained for this purpose, examined many sanitation amenities. These included a clean water supply, which was found to be absent. The presence or absence of latrines or sewage facilities was also noted. Additionally, the enumerators assessed the availability of a wastewater disposal facility or system and the presence or absence of garbage disposal facilities or rubbish bins. The behaviors evaluated among the household residents encompassed actions such as opening the bedroom window, opening the family window, engaging in home and yard cleaning activities, appropriately disposing of the feces produced by newborns and preschool-aged children in the restroom, as well as disposing of waste materials in their designated locations.

A healthy home environment can be characterized by several key features, including a clean ceiling, permanent and watertight walls, floor tiles, windows in the family room and bedrooms, adequate ventilation, a kitchen smoke hole, and overall good ventilation. The critical attributes of adequate sanitation encompass providing a hygienic water source, latrines or sewage facilities, the availability of wastewater disposal systems, and the provision of rubbish disposal facilities or bins. The significance of positive behaviors entails individuals residing in a household consistently engaging in actions such as regularly opening bedroom and family windows, maintaining cleanliness inside the premises and surrounding yard, appropriately disposing of fecal matter from newborns and preschool-aged children in designated latrine facilities, and ensuring the disposal of waste materials. Conversely, an unhealthy residential setting demonstrates qualities fundamentally contrary to those seen in a healthy family environment.

### Cookie consumption and health status obedience monitoring

The study involved monitoring the cookie consumption of participants through three weekly home visits and biweekly anthropometric data measurements at *posyandu*. During each household visit, proficient enumerators documented data related to food consumption, distribution of cookies, the number of remaining cookies, the consequences of cookie consumption, and the present health status. The researchers assessed the health condition of children by surveying mothers about symptoms such as cough, cold, fever, and other ailments. Additionally, they inquired about any potential adverse effects, such as diarrhea or constipation, that may have occurred after consuming cookies. Sure, participants in the intervention and control groups expressed dissatisfaction with the cookies provided during the second, third, and fourth home visits, including those consumed by other members of their respective households. Children declined the consumption of cookies because of experiencing ennui, encountering an unpleasant taste, or succumbing to a preference for sugary treats.

Nevertheless, as the enumerators encouraged the mothers to continue consuming cookies to enhance their children’s desire, all participants subsequently developed a preference for consuming the cookies. Conversely, it was observed that two participants in the control group experienced symptoms of cough, fever, and cold for the 4-week trial. A combined total of eight subjects withdrew from participation in both experimental groups for the test. Within the intervention group, five participants discontinued their involvement in the trial. Specifically, two individuals were removed from the study due to their mothers experiencing exhaustion, two others owing to illness, and one participant expressed dissatisfaction with the sweet taste of the cookies. Two participants from the control group withdrew from the study due to self-reported feelings of boredom associated with the consumption of cookies (Figure 1).

### Procedure for making cookies

The cookies were prepared using a combination of potato starch, almond flour, and marmalade, including chicken eggs, powdered white sugar, a small quantity of wheat flour, butter, and pandanus/orange/strawberry extracts for flavoring. The tools utilized to produce the cookies included:

- A mixer.
- A digital oven.
- Cookie molds.
- A digital cookie scale.
- A rolling pin.
- Plastic materials for cookie packaging.

**Figure 2:**
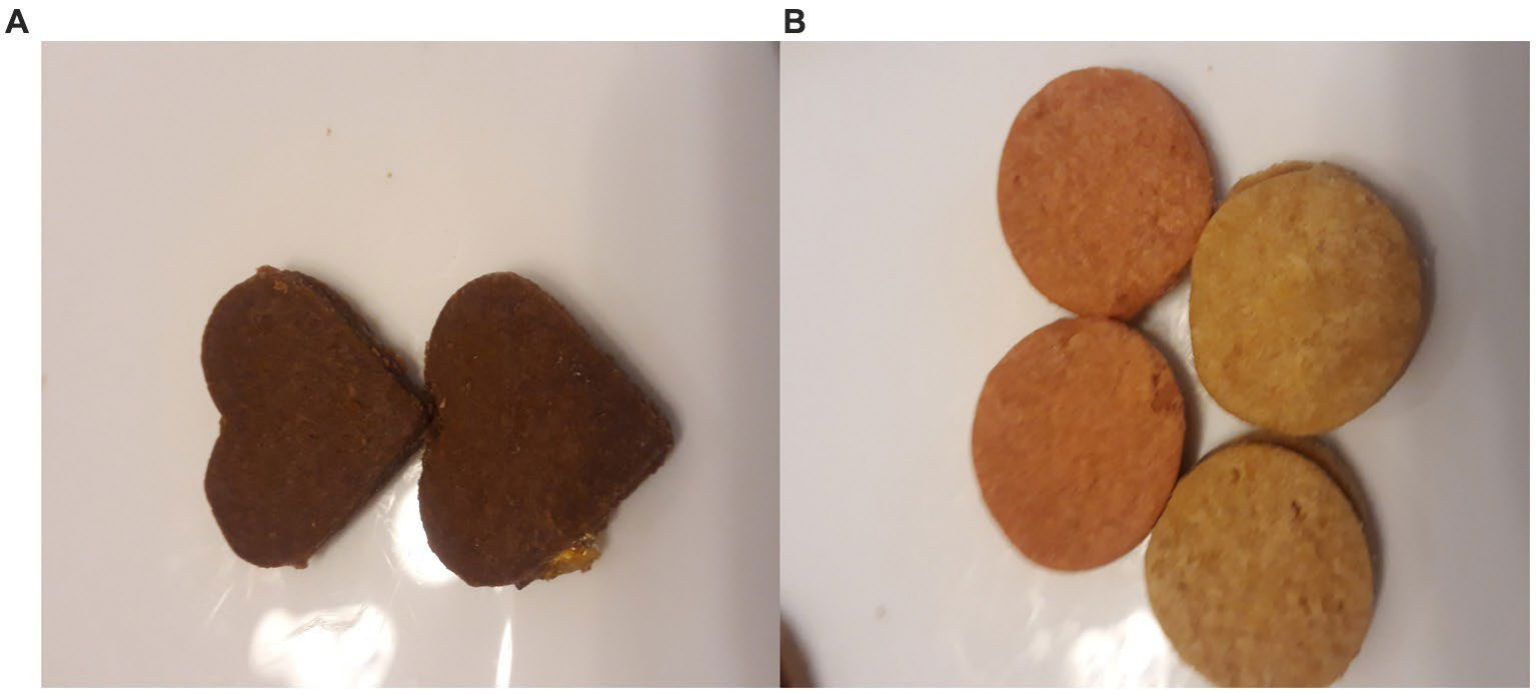
Almond orange potato cookie **(A)** and orange potato cookie **(B)**.

The initial stage in creating the cookies involved measuring the components using weighing. The butter was subjected to mixing with a mixer until it achieved a white appearance. Subsequently, the chicken eggs were incorporated into the dough by mixing. Subsequently, the pasta/flavor combination of chocolate, pandanus, and strawberry was incorporated and well-blended until a uniform hue was attained. Later, the potato starch, almond flour, and white sugar flour were mixed gradually and blended using a spatula until a uniform texture was reached. The dough was flattened and thinned using a rolling pin until smooth. After that, the dough was shaped using a cookie mold, and the resulting uncooked cookies were arranged on a sizable baking tray. The cookies were then subjected to a baking process in an oven set at a temperature of 180°C for 30 min. After being prepared, the cookies underwent a process of air cooling, followed by placing a layer of marmalade between two individual cookies. Subsequently, the completed cookies were enclosed in plastic packaging. Each bag of cookies weighed 50 g, as shown in Figure 2. The analysis of the nutrient composition, including macro- and micro-nutrients, of the cookies was conducted at the Saraswanti Genetech Laboratory located in Bogor City, Indonesia.

### Data analysis

The assessment encompassed evaluating the degree of cleanliness within the household, which contained several aspects such as the condition of housing components, sanitation facilities, and the inhabitants’ behaviors. The scores were determined using a ed. scoring methodology, wherein the values assigned to home components were multiplied by a factor of 31, sanitation facilities by a factor of 25, and resident behaviors by a factor of 44. The ultimate score was determined by aggregating the outcomes of the three evaluations. A home environment is acceptable if the overall score ranges from 1,068 to 1,200. Conversely, a total score below 1,068 indicates a hostile home environment. The study involved the comparison of maternal knowledge on nutrition and COVID-19, along with macro and micro-nutrients. The mean values of these variables were observed in both the treatment and control groups before and after the study. Values below the mean are categorized as insufficient, and values equal to or more than the mean are classified as sufficient.

A univariate analysis was conducted on the independent/explanatory variables, precisely the socio-demographic characteristics of the children’s family. These characteristics include maternal marital status, maternal age, maternal occupation, maternal educational background, father’s occupation, number of members in the nuclear family, children’s age, and sex. The results of this analysis are presented in Table 2. The analysis was conducted with the SPSS software program. The anthropometric data of the children before and after the intervention were analyzed using the WHO Anthro 3.2 Program. The analysis of the food ingested during the 4-week study involved the utilization of the Nutrisurvey Program to evaluate the levels of macronutrients (specifically energy, carbs, proteins, and fats) as well as micronutrients (namely vitamin E, calcium, and zinc) both before and after the study. The Kolmogorov–Smirnov test was employed to assess the normality of the change in HAZ score data post-study, demonstrating conformity to a Gaussian distribution (*p* = 0.2). The bivariate analysis involved the utilization of the independent t-test to examine the variables, namely HAZ score, macronutrient and micronutrient intakes, total cookie consumption, knowledge of balanced nutrition for preschool-aged children with stunting, and knowledge of COVID-19. These variables were compared between the intervention and control groups.

**Table 2:** Sociodemographic characteristic.

Variable	Control		Intervention		Total	
	*n*	%	*n*	%	*n*	%
Characteristic of family						
Marital status						
Married	23	100.0	19	100.0	42	100.0
Age of mother (years old)						
Mean ± SD	35.2 ± 7.7		36.1 ± 7.7		35.6 ± 7.6	
20–29	5	21.7	5	26.3	10	23.8
30–39	14	60.9	6	31.6	20	47.6
40–50	4	17.4	8	42.1	12	28.6
Mothers’ education						
Graduated from junior high school or less	10	43.5	5	26.3	15	35.7
Graduated from senior high school	12	52.2	11	57.9	23	54.8
Academy/bachelor	1	4.3	3	15.8	4	9.5
Working status of mother						
No	17	73.9	17	89.5	34	81.0
Yes	6	26.1	2	10.5	8	19.0
Occupation of father						
Freelance daily worker	11	47.8	8	42.1	19	45.2
Private employee/trader	12	52.2	11	57.9	23	54.8
Family size						
Nuclear family	15	65.2	15	78.9	30	71.4
Extended family	8	34.7	4	21.1	12	28.6
Characteristic of preschool-aged children						
Sex						
Female	14	60.9	11	57.9	25	59.5
Male	9	39.1	8	42.1	17	40.5
Age (month)						
Mean ± SD	30.4 ± 11.6		29.1 ± 10.9		29.8 ± 11.2	
12–24	7	30.4	9	47.4	16	38.1
25–36	10	43.5	5	26.3	15	35.7
37–48	3	13.0	4	21.1	7	16.7
49–59	3	13.0	1	5.3	4	9.5

## Results

Table 2 displays the socio-demographic characteristics of all participants, categorized into two distinct categories. All participants in this study who were mothers of preschool-aged children were married. It is worth noting that the intervention group’s mean age was slightly higher than the control group. The age group of 30–39 years constituted the most significant proportion of mothers. Most women had completed their education at the senior high school level. Still, a subset had attained higher levels of education, such as graduation from a college or university with a bachelor’s degree. Most mothers with preschool-aged children in both cohorts were not employed.

In contrast, most fathers were engaged in occupations as tradesmen and employees of private companies. The study observed a higher participation rate among females compared to boys. Additionally, the mean age of preschool-aged children in the intervention group was slightly lower than that of the control group. Most preschool-aged children in the intervention group were 12–24 months, whereas the preschool-aged children in the control group were predominantly between 25 and 36 months old (Table 2).

Table 3 presents a visual representation of the disparities in alterations of HAZ score concerning various socio-demographic attributes of mothers and children, as well as their awareness of balanced nutrition for preschool-aged children with stunting and knowledge of COVID-19, among both study groups. Based on the t-independent statistical test findings, a statistically significant disparity in the change of HAZ scores was detected when comparing the period before and after the consumption of orange almond potato cookies (*p* = 0.001). Many socio-demographic variables of mothers and preschool-aged children influenced HAZ scores within the intervention group. These characteristics included maternal age, education, father’s occupation, and cohabitation within the same household. Mothers who were at least 35 years old and had children with stunted growth were found to have a greater likelihood of experiencing significant increases in their HAZ score compared to mothers who were younger than 35 years old. Preschool-aged children whose mothers had adequate or secondary-level education exhibited a more pronounced alteration in their HAZ score compared to children whose mothers had a lower level of education. The study found that children under five, whose fathers were employed in private companies or trading exhibited a more significant growth in their HAZ score compared to children whose fathers worked as laborers or were involved in informal work. Preschool-aged children hailing from nuclear households consisting of three persons, without any siblings, had a more significant growth in their HAZ scores compared to those belonging to larger family units comprising a father, mother, children, and additional siblings or other relatives. There was no observed impact on the changes in Height-for-Age Z-score (HAZ) among both groups, regardless of their knowledge of balanced nutrition for preschool-aged children with stunting and knowledge of COVID-19 following a 4-week intervention.

**Table 3:** Change in the HAZ score difference based on the sociodemographic characteristics of mothers and stunting of children under-5 years old, balanced nutrition knowledge of stunting children under-5 years old, COVID-19 knowledge, healthy housing and sanitation, and intake of macro–micro nutrients in the two groups.

Variable	HAZ change						95% CI of difference	*p*
	Control			Intervention				
	*n*	Mean	*SD*	*n*	Mean	*SD*		
HAZ score change	19	0.5	0.2	23	0.3	0.2	0.1 to 0.4	*0.001
Total consumption of cookies (g)	23	713.5	368.6	19	800.5	406.9	−306.8 to 177.7	*0.03
Mothers’ age (y.o.)								
<35	9	0.3	0.2	8	0.4	0.9	−0.2 to 0.1	0.6
≥35	14	0.2	0.3	11	0.6	1.0	0.2 to 0.6	*0.001
p* (95% CI of difference)	0.2 (−0.1 to 0.3)			*0.02 (0.0 to 0.4)				
Mothers’ education level								
Low	10	0.2	0.2	5	0.4	0.2	−0.4 to 0.1	0.1
Medium	13	0.3	0.2	14	0.6	0.2	0.1 to 0.4	0.008
p* (95% CI of difference)	0.2 (−0.3 to 0.1)			0.1 (−0.5 to 0.0)				
Fathers’ job								
Informal sector workers	11	0.3	0.2	8	0.4	0.3	−0.3 to 0.1	0.4
Formal sector (private employee)	12	0.2	0.3	11	0.6	0.2	0.2 to 0.6	*0.001
* (95% CI of difference)	0.1 (− 0.0 to 0.4)			0.2 (−0.4 to 0.1)				
Family size								
Extended family	8	0.4	0.10	4	0.6	0.2	0.0 to 0.3	*0.048
Nuclear family	15	0.2	0.26	15	0.5	0.2	0.1 to 0.5	*0.002
* (95% CI of difference)	*0.0 (0.0 to 0.4)			0.7 (−0.2 to 0.3)				
Mothers’ nutritional knowledge level at pre-study								
Low	8	0.2	0.3	10	0.5	0.3	−0.5 to 0.1	0.1
Adequate	15	0.3	0.2	9	0.6	0.2	0.1 to 0.5	*0.001
* (95% CI of difference)	0.7 (−0.3 to 0.2)			0.2 (−0.4 to 0.1)				
Mothers’ nutritional knowledge level at post- study								
Low	7	0.3	0.3	10	0.5	0.2	−0.5 to 0.1	0.112
Adequate	16	0.2	0.2	9	0.5	0.3	0.1 to 9.5	*0.008
* (95% CI of difference)	0.5 (−0.1 to 0.3)			0.7 (−0.2 to 0.3)				
Mothers’ COVID knowledge level at pre-study								
Low	6	0.2	0.3	10	0.5	0.3	−0.6 to 0.1	*0.095
Adequate	17	0.3	0.2	9	0.5	0.2	0.1 to 0.4	*0.005
* (95% CI of difference)	0.8 (−0.3 to 0.2)			0.9 (−0.2 to 0.3)				
Mothers’ COVID knowledge level at post-study								
Low	10	0.3	0.3	10	0.54	0.24	0.0 to 0.6	*0.027
Adequate	13	0.2	0.2	9	0.47	0.24	0.0 to 0.4	*0.020
* (95% CI of difference)	0.1 (−0.2 to 0.2)			0.5 (−0.16 to 0.31)				

*p < 0.05. Independent *t*-test.

The characteristics of a healthy home environment are outlined in Table 4. The study found that preschool-aged children residing in a conducive home environment had a more substantial improvement in their HAZ score than those living in an unfavorable home environment (*p* = 0.008). Significant disparities were observed in the alterations of the HAZ score between the two groups categorized by their access to sanitary facilities (*p* < 0.05). There was no significant association between the conduct of individuals residing in the household and changes in the HAZ score, as determined by the group analysis (*p* > 0.05). The presence of a healthy home environment resulted in significant changes in the HAZ score for both groups (*p* = 0.004). Moreover, the consumption of micronutrients, specifically calcium and zinc, impacted the HAZ score alterations among the treatment and control cohorts. The preschool-aged children in the intervention group with sufficient calcium and zinc demonstrated a more pronounced alteration in the HAZ score than the control group. However, a contrasting scenario was observed with vitamin E intake. Preschool-aged children in the control group with sufficient carbohydrate intake exhibited a more significant alteration in the HAZ score than those with insufficient carbohydrate intake.

**Table 4:** Change in the HAZ score difference based on the healthy housing environment and sanitation, and intake of macro–micro nutrients in the two groups.

Variable	HAZ score change						95% CI of difference	*p*
	Control			Intervention				
	*n*	Mean	*SD*	*n*	Mean	*SD*		
Housing components								
Unhealthy	8	0.2	0.2	6	0.5	0.3	−0.6 to 0.0	0.075
Healthy	15	0.3	0.3	13	0.5	0.2	0.1 to 0.4	0.008
p* (95% CI of difference)	0.6 (−0.3 to 0.1)			0.8 (−0.3 to 0.2)				
Sanitary facilities								
Unhealthy	9	0.2	0.2	9	0.4	0.3	0.0 to 0.5	0.029
Healthy	14	0.3	0.3	10	0.6	0.2	0.0 to 0.5	0.006
p* (95% CI of difference)	0.2 (−0.3 to 0.1)			0.1 (−0.4 to 0.1)				
Occupant behavior								
Unhealthy	7	0.2	0.2	6	0.5	0.3	−0.6 to 0.1	0.102
Healthy	16	0.3	0.3	13	0.5	0.2	0.1 to 0.4	0.005
p* (95% CI of difference)	0.3 (−0.3 to 0.2)			0.6 (−0.3 to 0.2)				
Residential sanitation								
Unhealthy	7	0.2	0.2	6	0.5	0.3	−0.6 to 0.1	0.094
Healthy	16	0.3	0.3	13	0.5	0.2	0.1 to 0.4	0.005
p* (95% CI of difference)	0.6 (−0.3 to 0.2)			0.6 (−0.3 to 0.2)				
Energy intake level								
Low	9	0.3	0.2	12	0.5	0.2	0.0 to 0.4	0.029
Adequate	14	0.2	0.3	7	0.5	0.2	0.0 to 0.5	0.034
p* (95% CI of difference)	0.5 (−0.2 to 0.3)			0.8 (−0.2 to 0.3)				
Protein intake level								
Low	10	0.2	0.2	11	0.6	0.2	0.1 to 0.5	0.002
Adequate	13	0.3	0.3	8	0.4	0.3	−0.4 to 0.1	0.142
p* (95% CI of difference)	0.8 (−0.2 to 0.2)			0.3 (−0.1 to 0.4)				
Fat intake level								
Low	13	0.2	0.2	8	0.5	0.2	0.1 to 0.5	0.012
Adequate	10	0.3	0.3	11	0.5	0.3	0.1 to 0.5	0.045
p* (95% CI of difference)	0.5 (−0.3 to 0.1)			0.7 (−0.3 to 0.2)				
Carbohydrate intake level								
Low	9	0.2	0.3	12	0.5	0.3	0.1 to 0.5	0.024
Adequate	14	0.3	0.2	7	0.5	0.2	0.1 to 0.5	0.019
p* (95% CI of difference)	0.7 (−0.3 to 0.2)			0.8 (−0.3 to 0.2)				
Vitamin E intake level								
Low	12	0.3	0.2	10	0.5	0.3	−0.5 to 0.0	0.070
Adequate	11	0.2	0.2	9	0.5	0.1	0.1 to 0.5	0.004
p* (95% CI of difference)	0.4 (−0.1 to 0.3)			0.9 (−0.2 to 0.2)				
Calcium intake level								
Low	12	0.2	0.3	9	0.5	0.3	0.0 to 0.5	0.024
Adequate	11	0.3	0.2	10	0.5	0.2	0.0 to 0.4	0.018
p* (95% CI of difference)	0.2 (−0.3 to 0.1)			0.6 (−0.3 to 0.2)				
Zinc intake level								
Low	11	0.3	0.3	8	0.6	0.3	0.1 to 0.6	0.017
Adequate	12	0.2	0.2	11	0.5	0.2	0.0 to 0.4	0.023
p* (95% CI of difference)	0.9 (−0.2 to 0.2)			0.2 (−0.1 to 0.3)				

*p < 0.05. Independent *t*-test.

## Discussion

Non-anthropogenic calamities, such as the COVID-19 pandemic, have escalated the prevalence of stunting among preschool-aged children in Indonesia. The COVID-19 pandemic resulted in a disruption of the food supply chain, which in turn led to a rise in the prevalence of stunting among preschool-aged children (23, 26). Preschool-aged children who experience stunting are more susceptible to COVID-19 due to diminished immune levels stemming from inadequate consumption of macro- and micronutrients. To address this issue proactively, a targeted nutritional intervention was implemented to mitigate the primary factors contributing to growth impairment in children of preschool age. This intervention involved the provision of orange almond potato cookies as a dietary supplement for 4 weeks (27).

In the current investigation, the intervention group that consumed orange almond potato cookies had the most significant elevation HAZ score. Additionally, following 1 month of supplementation with orange almond potato cookies, the intervention group experienced a mean change in the HAZ score of 0.51. In contrast, the control group had a mean difference of 0.25. The potential for more considerable changes in HAZ score within the intervention group may be observed if the consumption of cookies extends beyond 1 month. The abovementioned observations are consistent with the outcomes of a research investigation on the effects of eel biscuit supplementation in preschool-aged children experiencing stunting in Bandung, Indonesia (28). Additionally, a separate study examining the impact of malalugis fish (*Decapterus macarellus*) meal and vermicelli cookies supplementation in Minahasa Regency, Indonesia (29), as well as another study investigating the effects of snail biscuit supplementation over 1 month in preschool-aged children with stunting in Surabaya, Indonesia (30), have yielded similar findings.

The intervention group’s HAZ score exhibited variations impacted by maternal age, maternal education, father’s occupation, and family size. According to a study, it has been shown that mothers belonging to the late adulthood age group (36–45 years) had a higher level of knowledge and maturity compared to those in the early adulthood age group (26–35 years) (31). This discovery presents a contradiction to the results obtained in the eel biscuit supplementation study conducted by Herawati et al. (28), wherein no statistically significant variations were observed among preschool-aged children with varying family characteristics, including maternal age, paternal age, mother’s occupation, and father’s occupation (32). The level of education attained by mothers is a significant factor in determining their ability to comprehend and acquire nutrition knowledge (33, 34). According to existing research (35), fathers employed in the private sector or engaged in trading tend to have higher income levels than laborers or informal workers. This disparity can be attributed to their permanent employment’s more excellent income stability. Their family size indirectly influences the efficacy of dietary interventions in preschool-aged children with stunting. Families who have more than two children or include extended family members may experience reduced food availability in situations where the family is economically disadvantaged (36).

The orange almond potato cookies exhibit higher levels of energy, protein, fat, zinc, calcium, and vitamin E than the almond potato cookies, as stated in Iannotti et al. (37). These six categories of nutrients play a significant role in promoting growth and development in preschool-aged children experiencing stunting. Variations in the alteration of HAZ scores were seen concerning the dietary levels of fat, calcium, and zinc. The rise in HAZ score is correlated with the consumption of fat, calcium, and zinc within the nutritional composition of cookies. This assertion is corroborated by previous research, which demonstrates that nutritional interventions incorporating fat, calcium, and zinc can potentially enhance the stature of children in the preschool age group (38–40). The presence of essential fatty acids in fat is necessary for promoting body growth, contributing to an increase in height (41). Potatoes’ energy content and carbohydrate composition have been found to be sufficiently high to contribute to gain (17, 19). Almonds, known for their significant vitamin E content, are also implicated in contributing to the growth in stature among preschool-aged children (41). Vitamin C in marmalade has been found to enhance the process of collagen production, leading to an eventual increase in bone density (42). A prior investigation on zinc supplementation in school-aged youngsters has also indicated that zinc plays a crucial role in promoting growth and augmentation (43). Zinc influences development by modulating various physiological processes, including DNA replication, immune system function, hunger regulation, and the production of growth hormones. These factors play crucial roles in the development and overall growth of the human body (44). Calcium assumes an essential function as a primary structural constituent in bone development. Consequently, insufficient calcium will impact children’s growth in the preschool age group (45, 46).

This current study does not possess the capability to establish a causal relationship between the knowledge of balanced nutrition and awareness about COVID-19 among parents of preschool-aged children with stunting and its impact on the HAZ score. This finding contradicts the results of previous research conducted in Yogyakarta, Indonesia (47), and Western Ethiopia (48), which examined the impact of maternal education level on stunting among preschool-aged children. The observed discrepancy could be attributed to variations in the duration of the intervention, the educational approach employed, and the size of the sample population. Mothers who possess a comprehensive understanding of balanced nutrition and have preschool-aged children are more likely to possess the ability to identify different nutritional diseases that contribute to stunting. Consequently, they can adapt their parenting practices, specifically in terms of feeding, daily care, and personal hygiene, to mitigate the occurrence of such illnesses. The acquired knowledge can be utilized in everyday life to alleviate the prevalence of dietary problems (49). The provision of sanitation amenities in residential households, including access to clean water, latrines or sewage systems, wastewater management systems, and garbage disposal facilities or receptacles, is associated with alterations in the HAZ score of preschool-aged children. According to two studies conducted in Indonesia, adequate sanitary facilities played a significant role in decreasing the prevalence of stunting among children in the preschool age group. The studies reported 29 and 41% reductions in stunting rates (50, 51). Promoting a conducive domestic setting is also a contributing factor in elevating HAZ score among preschool children who experience stunted growth. Hence, enhancing the domestic setting has the potential to mitigate the prevalence of stunting in preschool-aged children since an unsanitary home environment might contribute to diarrhea in this demographic, ultimately resulting in stunting (52).

### Limitations of the study

The subjects continually exhibit an interest in consuming cookies of various forms, such as heart and round, owing to the weekly variations in flavor, color, and look. The following procedures are employed to mitigate sensations of ennui and uphold enduring levels of engagement. The present study is subject to various limitations, including a small sample size, a brief intervention duration, and potential challenges in generalizing the findings to stunted children residing in different locations. The study was done in Indonesia and it may not translate well to stunned children living elsewhere.

The study’s intervention period they have spanned 1 month, during which it was observed that the intervention group saw a mean rise of 0.51 in their HAZ score. While the current intervention period lasts only 1 month, it is more probable that significant improvements in the HAZ score will be observed if the intervention period is prolonged beyond 1 month. Additional research is required to get optimal outcomes in assessing the potential for Indonesian stunted children to transition from a malnourished state to a normal nutritional status. This necessitates conducting more investigations with a more extensive sample size and an extended intervention duration of at least 3 months. Due to the limited time of the follow-up period in this study, our ability to evaluate the long-term viability of implementing supplemental feeding based on the intervention provided, as well as the extent to which mothers exhibited creativity and motivation in modifying almond orange almond potato cookies into alternative supplementary food items, was constrained. Furthermore, it is worth noting that as the sample size increases, the efficacy of the randomized technique in mitigating the influence of confounding variables also improves. The precision of the results will increase as more samples are collected.

### Areas for further research

The findings of this study have important implications for the Health District Office of Depok, particularly for policymakers. These implications can inform the development and implementation of successful nutritional intervention strategies in the community. One potential strategy that might be considered is the provision of nutrient-dense cookies to address the issue of stunted growth in children. Furthermore, to enhance food security and mitigate the prevalence of stunting among children under five in the post-pandemic period. Existing literature indicates a requirement for additional empirical data to assess the effectiveness of multi-sector interventions that incorporate both nutrition-specific and nutrition-sensitive approaches and programs. Furthermore, there is a need to examine the influence of “upstream” practices, policies, and initiatives implemented by governmental bodies, private sector entities, and non-governmental organizations on nutrition-related outcomes, particularly about stunting.

## Conclusion

The impact of a 4-week dietary intervention involving the provision of orange almond potato cookies to preschool-aged children with stunting is observed in the HAZ score, which shows a significant rise of 0.51. However, it is essential to note that the children’s stunting condition persists despite this improvement. The HAZ score of preschool-aged children with stunting who consume orange almond potato cookies is subject to direct influence from the cookies’ fat, calcium, and zinc content. Additionally, the sociodemographic characteristics of these children, including maternal age, maternal educational background, father’s occupation, nuclear family members, sanitation, and the presence of a healthy home environment, indirectly impact changes in their HAZ score. While there appears to be no significant impact on changes in HAZ score from parental knowledge of balanced nutrition and awareness of COVID-19 in preschool-aged children with stunting, it is still necessary to offer nutritional education alongside the provision of nutritional supplementation, such as orange almond potato cookies, to enhance adherence to consumption.

Further research is required to investigate the potential efficacy of supplementation in improving the nutritional status of under preschool-aged children and preschool-aged children with stunting. These studies should have a longer duration of at least 3 months and involve a larger sample size of at least 50 children. The objective is to explore the feasibility of utilizing supplementation to transition from a stunted nutritional state to a normal one.

## Data availability statement

The original contributions presented in the study are included in the article/supplementary material, further inquiries can be directed to the corresponding author.

## Ethics statement

The studies involving humans were approved by the Ethics Commission for Health Research and Development (KEPPK) of the Sint Carolus School of Health Sciences, Jakarta (Ethical Clearance No.008/KEPPKSTIKSC/I/2023), http://ClinicalTrials.gov Identifier: NCT05889819 https://clinicaltrials.gov/ct2/results?cond=&term=NCT05889819. The studies were conducted in accordance with the local legislation and institutional requirements. Written informed consent for participation in this study was provided by the participants’ legal guardians/next of kin. Written informed consent was obtained from the minor(s)’ legal guardian/next of kin for the publication of any potentially identifiable images or data included in this article.

## Author contributions

FF was involved in research idea conception, cookies formulation and development, study design, data collection and analysis, and manuscript writing. SU participated in the data analysis and manuscript review. All authors contributed to the article and approved the submitted version.
